# Red and Blue Light Affect the Formation of Adventitious Roots of Tea Cuttings (*Camellia sinensis*) by Regulating Hormone Synthesis and Signal Transduction Pathways of Mature Leaves

**DOI:** 10.3389/fpls.2022.943662

**Published:** 2022-07-07

**Authors:** Yaozong Shen, Kai Fan, Yu Wang, Hui Wang, Shibo Ding, Dapeng Song, Jiazhi Shen, He Li, Yujie Song, Xiao Han, Wenjun Qian, Qingping Ma, Zhaotang Ding

**Affiliations:** ^1^Tea Research Institute, Qingdao Agricultural University, Qingdao, China; ^2^Rizhao Tea Research Institute, Rizhao, China; ^3^Tea Research Institute, Shandong Academy of Agricultural Sciences, Rizhao, China; ^4^College of Agronomy, Liaocheng University, Liaocheng, China

**Keywords:** *Camellia sinensis* (L.) O. Kuntze, short cutting, adventitious root formation, WGCNA, phytohormone, plant hormone signal transduction, phytohormone transport, light quality

## Abstract

Light is an important environmental factor which affects plant growth, through changes of intensity and quality. In this study, monochromatic white (control), red (660 nm), and blue (430 nm) light-emitting diodes (LEDs) were used to treat tea short cuttings. The results showed the most adventitious roots in blue light treated tea cuttings, but the lowest roots in that treated by red light. In order to explore the molecular mechanism of light quality affecting adventitious root formation, we performed full-length transcriptome and metabolome analyses of mature leaves under three light qualities, and then conducted weighted gene co-expression network analysis (WGCNA). Phytohormone analysis showed that Indole-3-carboxylic acid (ICA), Abscisic acid (ABA), ABA-glucosyl ester (ABA-GE), trans-Zeatin (tZ), and Jasmonic acid (JA) contents in mature leaves under blue light were significantly higher than those under white and red light. A crosstalk regulatory network comprising 23 co-expression modules was successfully constructed. Among them, the “MEblue” module which had a highly positive correlation with ICA (*R* = 0.92, *P* = 4e-04). KEGG analysis showed that related genes were significantly enriched in the “Plant hormone signal transduction (ko04075)” pathway. *YUC* (a flavin-containing monooxygenase), *AUX1*, *AUX/IAA*, and *ARF* were identified as hub genes, and gene expression analysis showed that the expression levels of these hub genes under blue light were higher than those under white and red light. In addition, we also identified 6 auxin transport-related genes, including *PIN1*, *PIN3*, *PIN4*, *PILS5*, *PILS6*, and *PILS7*. Except *PILS5*, all of these genes showed the highest expression level under blue light. In conclusion, this study elucidated the molecular mechanism of light quality regulating adventitious root formation of tea short cutting through WGCNA analysis, which provided an innovation for “rapid seedling” of tea plants.

## Introduction

Tea [*Camellia sinensis* (L.) O. Kuntze] is a beverage crop in the world (Wei et al., 2018). Tea plant propagation includes sexual and asexual propagation. Asexual propagation is mainly used in tea propagation, and short cutting is one of the most commonly used methods. Although short cuttings have the advantages of large propagation coefficient and good characters, most short cuttings are carried out under natural conditions, which have the disadvantages of long seedling raising cycle and slow emergence speed. This seriously hinders the breeding and popularization of excellent varieties. The formation of adventitious roots is an important part of short cuttings. Accelerating the formation of adventitious roots is very important for the “rapid seedling” of tea plants.

As an environmental factor, light has an important impact on plant growth and development. After a long period of evolution, plants have adapted to broad- spectrum of light in the natural environment. In plant photosynthesis, red light (650–760 nm) is the most absorbed by plants, followed by blue light (430–470 nm). Light is not only an energy source for plant photosynthesis, but also a regulator of plant physiological activity. Numerous studies have shown that light quality, light intensity, and photoperiod have extensive regulatory effects on plant morphogenesis, physiological metabolism, growth and development and nutritional quality (Anderson, 1999; Ward et al., 2005; Qian and Kubota, 2009; Pérez-Balibrea et al., 2010).

Using light quality to regulate plant growth and development is the most effective and energy-saving way to regulate the light environment, and it is the core of the formulation of light environment regulation strategies. At present, the effect of light quality on the growth and development of agronomic plants has been widely studied. For example, Lim and Eom (2013) showed that irradiation of *Ocimum bacilicum* cuttings with blue LEDs can significantly shorten the root development time compared to red LEDs and natural sunlight. In a study of de-rooted *Picea abies* seedlings, red light promoted the formation of adventitious roots (Alallaq et al., 2020). Other studies reported that blue light stimulated root formation in *Betula pendula* (Sæbø et al., 1995) and *Chrysanthemum* (Chan et al., 2020) cuttings. The above researches show that different plant species need different light quality. In addition, there are relatively few reports on the effects of light quality on the growth and development of tea plants, and the underlying mechanism of the effects of different light quality on the growth and development of tea plants is still unclear.

In recent years, plant factories have developed rapidly, and light-emitting diodes (LEDs) are the main light sources in plant factories. LEDs have the characteristics of small size, light weight, long life, high light efficiency and low energy consumption (Bula et al., 1991; Brown et al., 1995). LEDs have peak wavelengths from 250 nm (UV) to 1,000 nm (infrared) (Bourget, 2008). Also, it provides wavelengths (light quality) that match the spectral range required for plant growth (Massa et al., 2008; Yen and Chung, 2009). In addition, LEDs are “cold light sources,” which will not attract some pests that like light and heat, and also help to reduce plant diseases and insect pests. Therefore, LEDs were chosen as the light source for this experiment.

In this study, white (control), red (660 nm), and blue (430 nm) LED tubes were used to treat short cuttings of *C.* Jiukengzaoand culture for 90 days. It was found that the adventitious root formation was the best under blue light compared with white light, while the adventitious root formation was the worst under red light. However, the mechanism of how light quality regulates adventitious root formation remains unclear. Therefore, we first performed transcriptome and hormonal analysis of mature leaves under white, red, and blue light, and then used weighted gene co-expression network analysis (WGCNA) to identify co-expressed gene modules and hub genes for adventitious root formation. Finally, the expressions of hub genes and auxin transport-related genes under different light quality were analyzed to elucidate the regulatory mechanism of light quality on adventitious root formation, which provided a new idea for the “rapid seedling” of tea plants.

## Materials and Methods

### Plant Materials and Light Treatment

The experiment was conducted in the artificial climate chamber of Rizhao Tea Research Institute (Rizhao, Shandong, China, 35°514 ex 119°662 ex from June 21 to September 18, 2021. All LED tubes were purchased from Shenzhen Hongyang Lighting Co., Ltd. (Shenzhen, China). The short cuttings of *C.* Jiukengzao were placed in a seedling box with substrate soil (2:1 volume mixture with perlite) and exposed to white (control, W), red (660 nm, R), and blue (430 nm, B) LEDs for light treatment. The light intensity was 100 μmol m^–2^ s^–1^, day (16 h, 8:00 a.m.–23:59 p.m., 28°C) night (8 h, 0:00–7:59 a.m., 20°C), and air humidity 85 ± 5%. After 90 days of treatment, the mature leaves were harvested, and immediately frozen in liquid nitrogen, then stored at −80°C for transcriptome sequencing and phytohormone assays. Three biological replicates were performed under white, red, and blue light.

### Determination of Phenotypic and Physiological Characteristics of Tea Cuttings

At 90 days of treatment, the formation status of adventitious roots under different light quality were observed and photographed. Six cuttings under different light quality were randomly selected and the longest root was measured with a ruler of 0.1 cm. The Fv/Fm values of the mature leaves were measured using the FP110-LM/D instrument (PSI, Czech Republic), and then the Soil and plant analyzer development values (SPAD) and Nitrogen content (N content) of the mature leaves under white, red and blue light were measured using the TYS-4N plant nutrient meter (Zhejiang Topo Yunnong Technology Co., Ltd., China).

### Phytohormone Determination

There are no roots in the initial stage of cuttings, the phytohormones mainly come from the mature leaves. Therefore, in this study we mainly measured the phytohormone content of the mature leaves under 3 light qualities. The mature leaves under white, red and blue light were immediately frozen in liquid nitrogen and ground into powder (30 Hz, 1 min). Then, the hormone content was determined based on the QTRAP6500+LC-MS/MS platform, MetWare.^1^ All assays were performed with three biological replicates.

### cDNA Library Construction and Oxford Nanopore Technoligies RNA-Seq

Total RNA was isolated from mature leaves of differentially treated short cuttings using the RNAprep Pure Plant Kit (Tiangen, Beijing, China). Three biological replicates were included per treatment. RNA size and quality were assessed by 1.2% agarose gel electrophoresis and spectrophotometer (NanoDrop 200c, Thermo Fisher Scientific), followed by the Agilent Bioananlyzer 2100 (Agilent Technologies, Santa Clara, CA, United States) and RNA Analysis Kits to assess whether the RNA has been degraded. After RNA quality verification, 9 cDNA libraries were constructed using a PCR-cDNA Sequencing Kit (SQK-PCS109) and then sequenced on the Nanopore PromethION plantform. Transcriptome analysis was performed by Biomarker Technologies Co., Ltd. (Beijing, China).

### Quality Control and Identification of Full-Length Transcripts

The Oxford Nanopore Technoligies (ONT) RNA-seq raw data were filtered (read quality > 6, read length > 350 bp) to remove low quality reads. rRNA was discarded after mapping to the rRNA database. Next, full-length non-chemical (FLNC) transcripts were identified by searching for primers at both ends of the reads. Clusters of FLNC transcripts were obtained after mapping to the reference genome of *Camellia sinensis* “Shuchazao” (CSS_ChrLev)^2^ with Mimimap2 and consensus isoforms were obtained after polishing within each cluster using the Pinfish package.^3^ Finally, the high-quality isoforms were mapped to the reference genome of tea plant. The reads were further folded and mapped by cDNA_ Cupcake package with minimum coverage = 85% and minimum recognition rate = 90%. The 5′ differences were not considered when folding the redundant transcripts. This resulted in full-length non-redundant transcripts.

### Identification of Differentially Expressed Genes and Annotation of Gene Function

The differentially expressed genes (DEGs) were identified from reads of nine cDNA libraries that were successfully aligned to the reference genome. Reads with a match quality higher than 5 were used for further quantification. Gene expression levels were estimated by counts per million. Differential expression analysis was performed on the mature leaves of tea cuttings under different light quality using the DESeq R software package. *P*-value ≤ 0.05 and | log_2_ Fold change| > 1 were set as thresholds for significant differential expression.

Gene function annotation was performed based on the following databases: NCBI non-redundant protein sequences (NR), protein families (Pfam), homology clusters of proteins (KOG/COG/eggNOG), manually annotated, and reviewed protein sequence database (Swiss-Prot), Kyoto Encyclopedia of Genes and Gene (KEGG) and Gene Ontology (GO).

### Weighted Gene Co-expression Network Analysis

Pearson correlation matrix and network topology analysis was used to calculate the genetic correlations of 9 samples using the R package WGCNA version 1.42 with the following settings: CPM value > 1, Fold change value > 1, minimum module size of 30, and minimum height of the merged modules of 0.055. Then, the adjacency matrix was transformed into a topological overlap matrix. A hierarchical clustering tree was constructed using R’s dynamic tree cutting package. The genes with KME > 0.8 in the correlation network were defined as hub genes.

### Validation of Oxford Nanopore Technoligies RNA-Seq by qRT-PCR

To verify the accuracy of the transcriptome data, 8 hub genes were selected for expression level validation. Primers were designed using Beacon Designer 8, and the primer sequences are shown in Supplementary Table 1. Quantitative real-time PCR (qRT-PCR) was performed on an analytikjena-qTOWER2.2 fluorescence quantitative PCR instrument (Germany) using 2 × SYBR^®^ Green master mix (DF, China). Three biological replicates were analyzed. Using the glyceraldehyde 3-phosphate dehydrogenase (*CsGAPDH*) gene as an internal reference gene, the relative expression was quantified by the 2^–ΔΔCt^ method.

### Statistical Analysis

Statistical analysis was performed using SPSS 20.0 software (SPSS Inc., Chicago, United States). One-way analysis of variance (ANOVA) and Duncan’s multiple intervals were used to analyze significant differences between physiological and hormonal index data under different light quality, and differences were considered statistically significant at *P*-values < 0.05. Graphics were created using Adobe Photoshop CC 2019.

## Results

### Differences of Adventitious Root Formation and Physiological Characteristics of Tea Cuttings Under Different Light Quality

**FIGURE 1:**
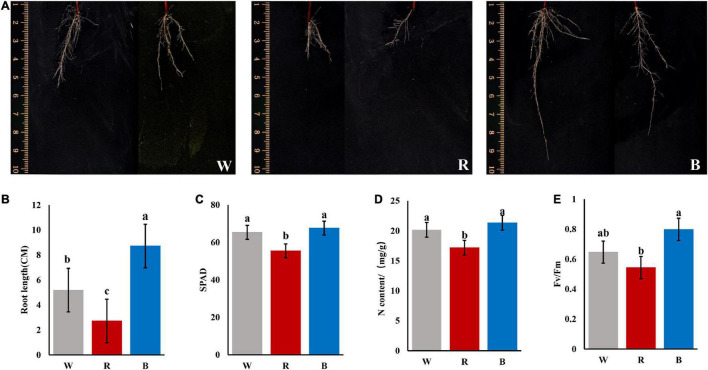
Phenotypic and physiological indicators of cuttings under different light quality after culturing for 90 days. **(A)** Root phenotypes of cuttings under 3 light qualities. **(B)** Longest root length (unit:CM). **(C)** SPAD value of mature leaves. **(D)** N contents of mature leaves (unit: mg/g). **(E)** Fv/Fm ratio of mature leaves (*P* < 0.05).

At 90 days of treatment, we carried out sampling statistics on the cuttings. Formation status of adventitious roots: blue light > white light > red light, the callus expanded but few roots under red light (Figures 1A,B). In conclusion, blue light can quickly induce the formation of adventitious roots.

Light quality has influences on plant leaf color, and the SPAD value is a parameter that measures the relative chlorophyll content of the plant, or indicates the degree of greenness of the plant. As shown in Figure 1C, the SPAD value under red light (55.483) was significantly lower than that under white light (65.317) and blue light (67.583). The N contents of mature leaves were also measured and the result was consistent with the pattern of variation of the SPAD values (Figure 1D).

Light quality has a great influence on photosynthesis in plants. Fv/Fm is the maximum photochemical quantum of PSII (optical/maximal photochemical efficiency of PSII in the dark), which reflects the efficiency of light energy conversion within the PSII reaction center or the potential maximum photosynthetic capacity of plants (photosynthetic efficiency). As shown in Figure 1E, Fv/Fm ratio: blue light (0.798) > white light (0.647) > red light (0.543).

### Change Patterns of Phytohormones in Mature Leaves Under Different Light Quality

**FIGURE 2:**
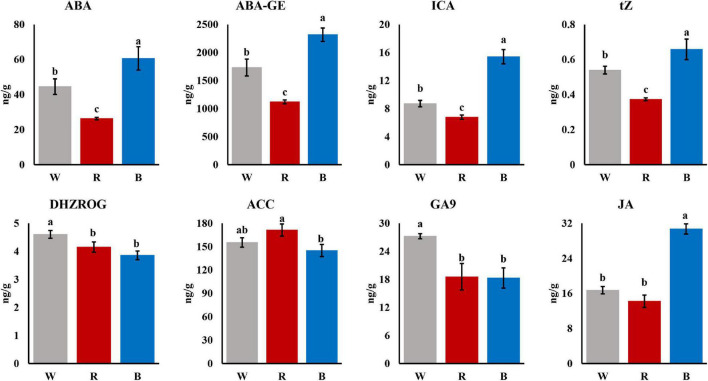
Phytohormones in mature leaves under 3 light qualities (*P* < 0.05).

To reveal the underlying mechanism of the differences in adventitious roots among different light quality, we measured the phytohormone contents of the mature leaves under different light quality. As shown in Figure 2, most of the phytohormones were significantly different under the 3 light qualities. Among them, five phytohormones, Abscisic acid (ABA), ABA-glucosyl ester (ABA-GE), Indole-3-carboxylic acid (ICA), trans-Zeatin (tZ), and Jasmonic acid (JA), showed an overall change pattern similar to that of root growth, showing blue light > white light > red light. In addition, there were significant differences in the ratios of certain phytohormones. For example, the ICA/DHZROG under blue light was significantly higher than that under white and red light (Supplementary Figure 1).

### Global Expression Analysis of Tea Plants Under Different Light Quality

To reveal the molecular mechanism of differences in adventitious root formation among different light quality, 9 samples were collected from 3 light qualities for ONT RNA-seq. A total of 52.11 Gb clean data were obtained by high-throughput sequencing. After removing low-quality reads, a total of 38.6 million clean reads were obtained, and the average number of reads per library was about 4.3 million. More than 92% of the reads could be mapped to the reference genome sequence (Supplementary Table 2).

**FIGURE 3:**
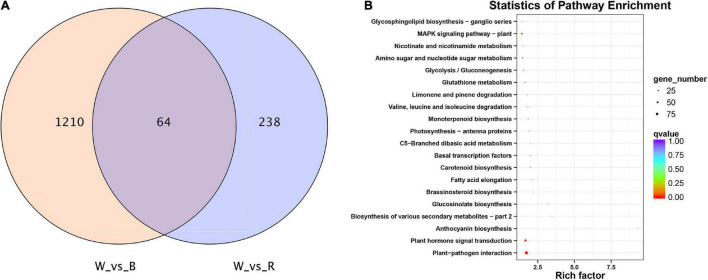
**(A)** VENN diagram showing the number of differentially expressed genes (DEGs) per comparison between different light quality in mature leaves. **(B)** Top 20 statistics of KEGG pathway enrichment in mature leaves under 3 light qualities.

A total of 1,512 DEGs were identified by red and blue light compared to white light. Among them, there are 64 common DEGs in red and blue light (Figure 3A). These genes were searched using KEGG to analyze their possible functions in adventitious root formation. The results showed that these genes were significantly enriched in “Plant hormone signal transduction (ko04075)”and “Plant-pathogen interaction (ko04626)” (Figure 3B). Above results suggested that these pathways may be involved in the regulation of adventitious root formation by light quality.

### Co-expression Network Analysis Between Different Light Quality

**FIGURE 4:**
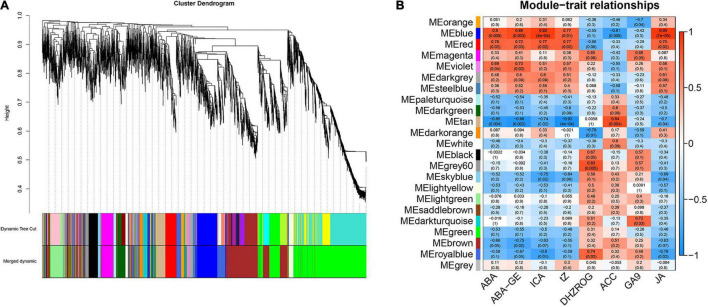
Weighted gene co-expression network analysis of the genes in mature leaves under 3 light qualities. **(A)** Hierarchical clustering tree representing co-expression modules identified by WGCNA. The main branches marked with different colors make up 23 modules. **(B)** WGCNA analysis between DEGs and phytohormones under 3 light qualities. Each row represents a module. The color and number of each cell represents the correlation coefficient between modules and phytohormones.

To explore the relationship between phytohormone content and gene expression, we constructed a co-expression network using WGCNA. Finally, a total of 23 gene co-expression modules (marked with different colors) were identified in the cluster dendrogram (Figure 4A). And the soft-thresholding of WGCNA was 5 (Supplementary Figure 2), this value proved to be reliable (Wu et al., 2021; Harutyunyan et al., 2022). The “MEblue” and “MEred” modules which had highly positive correlations with 5 kinds of phytohormones (*P* < 0.05), including ABA, ABA-GE, ICA, tZ, and JA, and the correlation coefficients were all greater than 0.7. Among them, the correlation coefficient between “MEblue” module and ICA is as high as 0.92. The “MEgrey60” and “Meroyalblue” modules which had highly positive correlations with DHZROG (*R* > 0.7, *P* < 0.05), the “MEtan” module had a highly positive correlation with ACC (*R* = 0.84, *P* = 0.004), and the “MEdarkturquoise” module had a highly positive correlation with GA9 (*R* = 0.72, *P* = 0.03). In addition, the “MEtan” module had highly negative correlations with ABA, ABA-GE, ICA, tZ, and JA (*R* < −0.7, *P* < 0.05), and the “MEroyalblue” module had highly negative correlations with ICA and JA (*R* < −0.75, *P* < 0.05) (Figure 4B).

**FIGURE 5:**
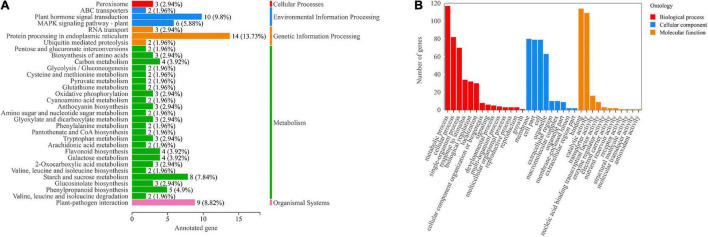
**(A)** KEGG pathway annotation of genes in the “MEblue” module. Numbers next to the bar graph indicate gene enrichment for this pathway. **(B)** GO annotations of genes in the “MEblue” module.

To further understand the functions of genes in the “MEblue” module, we performed KEGG and GO enrichment analyses. The results of KEGG enrichment analysis showed that most genes were enriched in the “Plant hormone signal transduction (ko04075),” “Plant-pathogen interaction (ko04626)” and “Starch and sucrose metabolism (ko00500)” pathways (Figure 5A). GO enrichment analysis showed that most genes were enriched in “metabolic processes,” “cellular processes” and “single-cell processes” in biological processes; most genes were enriched in “membranes,” “membrane components” and “cells” in cellular components; most genes were enriched in “combined” and “catalytic activity” in molecular functions (Figure 5B). The above results indicated that these pathways were highly correlated with phytohormones and played an important role in the regulation of adventitious root formation by light quality.

### Identification of Hub Genes and Auxin Transporter-Related Genes

**FIGURE 6:**
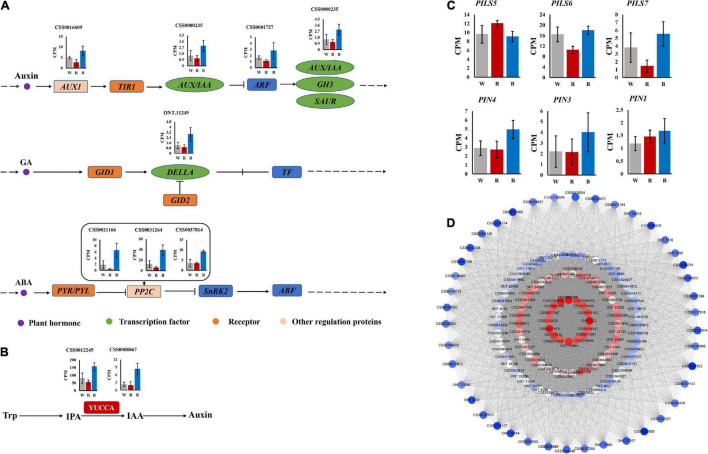
**(A)** Auxin, GA and ABA signaling pathways. **(B)** Auxin synthesis pathway. **(C)** Expression of auxin transport-related genes. **(D)** Correlation network graph of genes in “MEblue” module.

To gain insight into the regulatory mechanisms of phytohormone-related genes associated with adventitious root formation of the cuttings, hub genes with high connectivity in the network were selected based on KME values (KME > 0.8). The correlation network graph of some genes is shown in Figure 6D. The results showed that several genes were involved in auxin synthesis, transport and response, including 2 flavin monooxygenases *YUC* (a flavin-containing monooxygenase) (CSS0012245, CSS0008867), 1 auxin influx carrier *AUX1* (CSS0016609), 1 auxin early responsive protein *AUX/IAA* (CSS0000235), and 1 auxin response factor *ARF* (CSS0001727). In addition, *DELLA* protein (ONT.11249) involved in GA signaling, and *PP2C* (CSS0021166, CSS0031264, CSS0037814), a negative regulator of ABA signaling, were also identified as hub genes in the network (Supplementary Table 3).

The expression profiles of these hub genes were analyzed under 3 light qualities. As shown in Figures 6A,B, compared with white light, all genes were up-regulated under blue light, while most were down-regulated under red light. Among them, *YUC*, *DELLA*, and *PP2C* were more significantly up-regulated under blue light.

Additionally, we identified 6 auxin transport-related genes, including two major categories, *PIN* and *PIN-LIKES* (*PILS*). *PIN* includes *PIN1* (CSS0005048), *PIN3* (CSS0044702), and *PIN4* (CSS0006843). *PILS* includes *PILS5* (CSS0008291), *PILS6* (CSS0006341), and *PILS7* (CSS0041860) (Supplementary Table 4). Except for *PILS5*, which had the lowest expression under blue light, all others had the highest expression under blue light (Figure 6C).

### qRT-PCR Analysis

**FIGURE 7:**
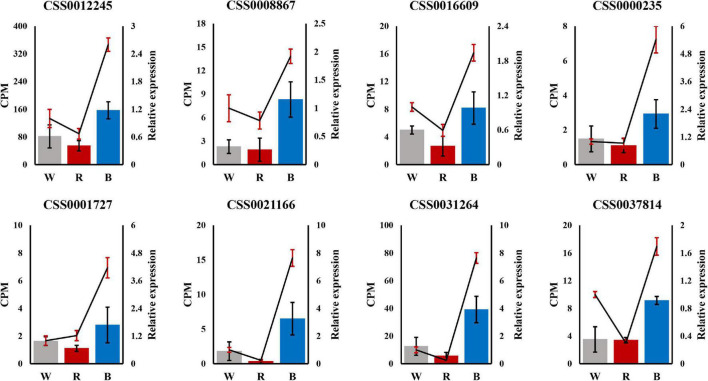
Validation of 8 hub genes by qRT-PCR. The left Y-axis represents counts per million (CPM) values for Oxford Nanopore Technoligies (ONT) RNA-Seq, and the right Y-axis represents relative qRT-PCR expression levels. In the figure, the bars represent ONT RNA-Seq, and the lines represent qRT-PCR.

To demonstrate the reliability of the ONT RNA-Seq data, we performed qRT-PCR to validate the expression profiles of the eight hub genes identified by WGCNA. Expression profiles between white and red light, and between white and blue light were compared by qRT-PCR. For all these selected genes, the expression trends by qRT-PCR were consistent with those of the ONT RNA-Seq data, which proved that the ONT RNA-Seq data were reliable (Figure 7).

## Discussion

### Red and Blue Light Affected Adventitious Root Formation by Regulating the Expression of YUC Genes

Previous studies reported that auxin is the master regulator of adventitious roots and plays a key role in the formation of adventitious roots in plants (Negishi et al., 2014; Kreiser et al., 2016; Pan et al., 2020). ICA belongs to auxin, and the content of ICA under blue light is significantly higher than that under red light (Figure 2). It has been reported that *YUC*, as a flavin monooxygenase, catalyzes the irreversible oxidative decarboxylation of indole-3-pyruvate acid (IPyA) to create IAA, which is the core of auxin formation (Zhao et al., 2001; Zheng et al., 2013), and up-regulation of *YUC* genes can promote IAA biosynthesis (Han et al., 2020). There are also reports certificated that the *YUC* gene family orchestrated the biosynthesis of endogenous auxin required for adventitious root induction (Chen et al., 2016; Sang et al., 2016). In addition, study has shown that *YUC6* is positively correlated with the formation of adventitious roots in Arabidopsis (Rovere et al., 2016). Meanwhile, Pan et al. (2019) showed that enhanced *YUC1* gene expression in mature leaves could rescue the rooting defects caused by leaf maturation Yamamoto et al. (2007) also demonstrated that antisense expression of *YUC* in rice inhibited root formation and elongation, similar to the root phenotype of auxin-insensitive mutants The above results indicated that *YUC* genes play an important role in auxin biosynthesis and adventitious root formation. Moreover, *YUC* genes are widely present and highly conserved in the plant kingdom (Liu et al., 2014). In the study of cucumber, the expression of *YUC* gradually increased with the adding of the proportion of blue light. In this study, as a hub gene, expression of *YUC* significantly increased under blue light compared with red light (Figure 6B). The higher expression of *YUC* gene augmented the content of ICA under blue light. Therefore, we speculated that blue light promotes the synthesis of ICA by up-regulating the expression of *YUC* gene, thereby promoting the formation of adventitious roots of cuttings.

### Red and Blue Light Affected Adventitious Root Formation by Regulating the Expression of Auxin Transport-Related Genes

During the induction phase of adventitious root formation, auxin was synthesized in the mature leaves and transported to the cutting base. In the study of Arabidopsis, light affects IAA synthesis during seedling germination, and IAA transport from developing leaves to roots (Bhalerao et al., 2002). Auxin transport is usually mediated by auxin influx and efflux vectors, which are encoded by the *AUX1/LAX*, *PIN*, and *PILS (PIN-LIKES)* genes, respectively (Zhao et al., 2021). Members of the *AUX1/LAX* gene family are major auxin influx carriers, and mutations in its gene cause auxin-related developmental defects and are involved in the regulation of key plant processes, including root, and lateral root development, root gravitation and hair development, vascular patterns, seed germination, leaf morphogenesis, and embryo development (Swarup and Bhosale, 2019). In this study, *AUX1* was also identified as a hub gene. It has been shown that *AUX1* promotes auxin transport between leaves and root tissues through the phloem, thereby regulating root development (Marchant et al., 2002). In Liriodendron hybrids, *AUX1* is highly expressed in adventitious roots, and the expression of *AUX1* and *ARF1* is increased during adventitious root development (Zhong et al., 2015), suggesting that *AUX1* plays an important role in adventitious root formation, *AUX1* and *ARF* may also play a synergistic role in regulating plant root development. In this study, *AUX1* and *ARF* were expressed at the highest levels under blue light, which proved that this synergistic effect may exist. In Arabidopsis, *PIN1* and *AUX1* coordinate to promote auxin flux, thereby inducing auxin levels sufficient to touch off adventitious root initiation. On the other hand, *LAX3* is also active during adventitious root formation (Rovere et al., 2016). In addition, Fan et al. (2021) showed that the *AUX1/LAX* family was considered to be the key gene for adventitious root formation by studying the cuttings of three varieties of tea with different rooting abilities. Meanwhile, Yousef et al. (2021) investigated the effect of different light qualities on graft union formation in grafted tomatoes and showed that the expression levels of both *AUX1* and *ARF* were higher in blue LEDs than in red LEDs and white fluorescence. In our study, the expression level of *AUX1* under blue light was higher than that under red light (Figure 6A). Based on the above results, we believed that *AUX1* plays a crucial role in the formation of adventitious roots in cuttings induced by blue light.

PIN protein acts as an auxin efflux carrier, and its polar orientation corresponds well with the direction of auxin movement, which indicates that PIN protein is mainly responsible for the asymmetric distribution of auxin in plants (Benková et al., 2003; Swarup and Bennett, 2003; Swarup and Péret, 2012). Also, *PIN i*s involved in plant growth and development by regulating auxin flow and distribution (Petrásek and Friml, 2009). Among them, *PIN1* and *PIN3* act as major auxin transport promoters, mediating the polar redistribution of auxin from stem to root (Blilou et al., 2005; Krecek et al., 2009; Adamowski and Friml, 2015). This indicates that *PIN1* and *PIN3* contribute to the transport of auxin from above ground to below ground, thereby regulating root growth. Other study has shown that the promoters of *PIN1* and *PIN4* bind *XAL2*/*AGL14* to upregulate the expression of *PIN1* and *PIN4*, which is necessary for the overproduction of auxin transport to roots and participates in auxin-mediated root development in a *PIN4*-dependent manner (Garay-Arroyo et al., 2013). In the study of maize, overexpression of *ZmPIN1a* was found to promote the transport of auxin from the stem to the root system, resulting in a well-developed root system with longer primary roots (Li et al., 2018). In the study of Arabidopsis, *AtPIN1* was found to play a role in auxin polar transport and promote auxin efflux from cells (Gälweiler et al., 1998). Another study reported that blue light-induced Arabidopsis adventitious root formation was regulated by *PIN3*-mediated auxin transport (Zhai et al., 2021). In this study, the formation of adventitious roots was the best under blue light, and *PIN1*, *PIN3* and *PIN4* were all expressed at the highest levels under blue light (Figure 6C), so we believe that *PIN1*, *PIN3*, and *PIN4* promoted the transport of ICA from mature leaves to the base of cuttings, and play a key role in the formation of adventitious root induced by blue light.

*PILS* are a newly discovered class of auxin transporters that assist the polar transport of auxin. PILS protein has certain similarity with PIN protein in protein sequence and contributes to the homeostasis of intracellular auxin (Feraru et al., 2012; Sun et al., 2020). Currently, little is known about the effects of PILS proteins on plant growth and development. Previous studies have shown that *PILS* transcription integrates environmental signals such as light and temperature to play an important role in the process of auxin-dependent growth, and affects de novo organogenesis and the growth rates of root and stem (Béziat et al., 2017; Feraru et al., 2019). In study of Arabidopsis, it was shown that *PILS2* and *PILS5* have redundant roles in regulating root growth, with significantly longer roots in *pils2* single mutant and *pils2 pils5* double mutant seedlings compared to wild type, and root systems in *PILS5* seedlings were shorter (Barbez et al., 2012). Additionally, *AtPILS1-7* in Arabidopsis has been shown to contribute to intracellular auxin transport (Barbez et al., 2012; Béziat et al., 2017; Feraru et al., 2019). In this study, *PILS6* and *PILS7* had the highest expression under blue light, while *PLS5* had the lowest expression under blue light (Figure 6C). So we think *PILS5,6,7* may play an important role in auxin transport, and then play an important role in formation of adventitious root induced by blue light.

### Red and Blue Light Affected Adventitious Root Formation by Regulating the Expression of Hormone Signal Transduction Genes

Auxin plays a crucial role in the formation of adventitious roots. Plants can quickly sense and respond to changes in auxin levels, which involves several types of auxin response factors, such as the Auxin/Indole-3-acetic acid (*AUX/IAA*) family, the auxin response factor (*ARF*) family, and the small auxin up-regulated RNA (*SAUR*) family (Luo et al., 2018). In this study, *AUX/IAA* and *ARF* were identified as hub genes. It has been reported that *IAA18* and *IAA28* transcripts can be transported from mature leaves to roots through the phloem to regulate root growth (Notaguchi et al., 2012), suggesting that *IAA* may have an important role in long-distance communication between leaves and roots. Studies have reported that *IAA17* is involved in some typical phenotypes controlled by auxin signaling, such as hypocotyl elongation, root gravitation, root hair, and adventitious root formation (Leyser et al., 1996; Rouse et al., 1998). Moreover, some studies have shown that *IAA* is also involved in lateral root formation by interacting with *ARF* proteins (Tatematsu et al., 2004; Sorin et al., 2005; Gutierrez et al., 2009), indicating that *IAA* and *ARF* have a synergistic effect in regulating plant root development. In our study, *AUX/IAA* and *ARF* were up-regulated under blue light, which also demonstrated the existence of this synergy. Previous studies have shown that *ARF6* and *ARF8* are positive response factors for adventitious root formation (Sorin et al., 2005; Gutierrez et al., 2009). Meanwhile, some studies have demonstrated that certain auxin signaling genes may play a role in both lateral root and adventitious root formation. For example, *ARF9* and *ARF17* can regulate lateral root and adventitious root formation in Arabidopsis (Okushima et al., 2005; Vanneste et al., 2005; Lee et al., 2009; Smet et al., 2010; Wilmoth et al., 2010). In Eucalyptus globulus, far-red light domestication of donor plants promoted adventitious root formation in cuttings compared with white light domestication, which was associated with higher expression levels of *ARF6* and *ARF8* during adventitious root formation (Ruedell et al., 2015), indicating that light quality can affect root formation by regulating the expression of *ARF.* In addition, single mutants of *arf7* and *arf19* reduced the number of lateral roots and adventitious roots, and the double mutants of *arf7* and *arf19* showed more obvious (Okushima et al., 2007; Wilmoth et al., 2010). Ma et al. (2021) treated tea seedlings with supplemental red light and found that *IAA26* was upregulated under red light. However, Ouyang et al. (2015) showed that *AUX/IAA* and ARF were significantly up-regulated in Norway spruce under blue light compared to red light. In our study, the expression of both *AUX/IAA* and *ARF* also was higher in blue light than in red light (Figure 6A). These results suggested that blue light may promote the formation of adventitious roots in cuttings by inducing the expression of *AUX/IAA* and *ARF* genes and coordinating their expression.

In addition to the auxin pathway, other hormone signal transduction pathways are also involved in the formation of adventitious roots in cuttings. *DELLA* and *PP2C* were also identified as hub genes in our study. *DELLA* proteins are negative regulators of GA signaling (Olszewski et al., 2002). In poplar, the expression level of *DELLA* gene increased continuously during adventitious root formation (Liu et al., 2016), indicating that *DELLA* plays an important role in adventitious root formation. The *PP2C* gene has been shown to be a negative regulator of *ABA* signaling (Park et al., 2009). The root elongation of the *TaPP2C-a10* transgenic line in Arabidopsis was not affected by high concentrations of ABA (Yu et al., 2020). In this study, ABA and ABA-GE contents were highest under blue light (Figure 2), but adventitious root formation was optimal, which may be attributed to the high expression of *PP2C*. Additionally, in a study by Zheng et al. (2019) that treated tea plants with blue and green light, *PP2C* was up-regulated under blue light, which is similar to our findings (Figure 6A). The above results indicated that *DELLA* and *PP2C* may also play a key role in the formation of adventitious roots in cuttings induced by blue light.

## Conclusion

In this study, the molecular mechanism of the difference in the formation of adventitious roots in tea plants under white, red, and blue light were investigated for the first time. The results showed that, compared with white light, blue light promoted the formation of adventitious roots, while red light showed inhibition. Phytohormone analysis showed that the content of ICA in mature leaves under blue light was significantly higher than that under white light and red light. Transcriptome results revealed that genes involved in auxin formation and signal transduction were expressed at different levels under different light qualities. The hub genes *YUC*, *AUX/IAA*, *ARF*, and *AUX1* were the key genes in the regulation of adventitious root formation in tea cuttings by light quality, and their expression levels were the highest under blue light. The auxin transport-related genes *PIN1*, *PIN3*, *PIN4*, *PILS6*, and *PILS7* showed the highest expression levels under blue light, while *PILS5* showed the lowest expression level under blue light. In conclusion, in actual production, certain amount of blue light could be supplied to promote the formation of adventitious roots of tea cuttings This study provides a new idea for the “rapid seedling” of tea plants in the future.

## Data Availability Statement

The raw data for RNA-seq have been uploaded to the NCBI SRA with accession number PRJNA851157.

## Author Contributions

YSh conducted an experiment, analyzed the data, and wrote a manuscript. HW, SD, and DS collected the samples. ZD and YW put forward hypotheses and designed experiments. JS, QM, and WQ reviewed the manuscript. HL, YSo, and XH participated in the experimental design and guided the research. All authors contributed to the article and approved the submission.

## Conflict of Interest

The authors declare that the research was conducted in the absence of any commercial or financial relationships that could be construed as a potential conflict of interest.

## Publisher’s Note

All claims expressed in this article are solely those of the authors and do not necessarily represent those of their affiliated organizations, or those of the publisher, the editors and the reviewers. Any product that may be evaluated in this article, or claim that may be made by its manufacturer, is not guaranteed or endorsed by the publisher.

## Footnotes

1
http://www.metware.cn/

2
http://tpia.teaplant.org/download.html

3
https://github.com/nanoporetech/pinfish
