# Supplementary material for: Red and Blue Light Affect the Formation of Adventitious Roots of Tea Cuttings (Camellia sinensis) by Regulating Hormone Synthesis and Signal Transduction Pathways of Mature Leaves
Source: Front Plant Sci. 2022 Jul 7;13:943662. doi: 10.3389/fpls.2022.943662 (PMC9301306; doi:10.3389/fpls.2022.943662)
Supplement: Supplementary file 1 [file Data_Sheet_1.docx]

**Table S1.** Primers used for real-time analysis. Primers used for real-time PCR analysis.

| Gene name | Genome code | Primer sequences (5’-3’) |
| --- | --- | --- |
| *YUC* | CSS0012245 | F: GATCAACTCTGCAAGGACAT  R: CCGTAAGGCTCAAACAACT |
| *YUC* | CSS0008867 | F: CAAAGCCCAGTTTTCCTAACC  R: CCATATAATCCTCTCCTTGCTAGT |
| *AUX1* | CSS0016609 | F: AGAGACCACCATCATTCCT  R: CCATCCTCCAAACCCAAAT |
| *AUX/IAA* | CSS0000235 | F: TGTGCCCACCTATGAAGATAA  R: TGCTTCAGATCCTTTCATTACG |
| *ARF* | CSS0001727 | F: TGATGTTCTACTTGTTGGG  R: CCTCTTCACTCATCTTCTG |
| *PP2C* | CSS0021166 | F: AGTGAGGCTGTAAATCAAAG  R: ATTCAAGTCCACTACTACCA |
| *PP2C* | CSS0031264 | F: GGTTGTTCAGGAGGTGTAT  R: CGGTTTCGTGTTCTATTCG |
| *PP2C* | CSS0037814 | F: AGATCCATGACTGATGATGAGGTT  R: GTTGTCTGAACTGTTTCTTCCGAA |
| *CsGAPDH* | KP053396 | F: TTGGCATCGTTGAGGGTCT  R: CAGTGGGAACACGGAAAGC |

**Table S2.** Overview of transcriptome data.

| Sample | Total Reads | Mapped reads | Mapped rates % |
| --- | --- | --- | --- |
| B-1 | 4,592,215 | 4,384,318 | 95.47% |
| B-2 | 4,702,090 | 4,501,626 | 95.74% |
| B-3 | 5,120,959 | 4,865,721 | 95.02% |
| R-1 | 4,587,183 | 4,363,108 | 95.12% |
| R-2 | 4,241,901 | 3,928,389 | 92.61% |
| R-3 | 4,213,927 | 3,964,325 | 94.08% |
| W-1 | 3,372,632 | 3,215,173 | 95.33% |
| W-2 | 4,477,441 | 4,247,226 | 94.86% |
| W-3 | 3,268,243 | 3,114,578 | 95.30% |
| Total | 38,576,591 |  |  |
| average | 4,286,288 |  |  |

**Table S3.** Annotation of phytohormone related hub gene

| Gene name | NR annotation | Name | KME |
| --- | --- | --- | --- |
| CSS0012245 | probable flavin-containing monooxygenase 1 [Camellia sinensis] | *YUC* | 0.900 |
| CSS0008867 | probable indole-3-pyruvate monooxygenase YUCCA10 [Camellia sinensis] | *YUC* | 0.940 |
| CSS0016609 | auxin transporter-like protein 3 [Camellia sinensis] | *AUX1* | 0.801 |
| CSS0000235 | auxin-responsive protein IAA14-like [Camellia sinensis] | *AUX/IAA* | 0.834 |
| CSS0001727 | hypothetical protein HYC85_008869 [Camellia sinensis] | *ARF* | 0.798 |
| ONT.11249 | scarecrow-like protein 15 [Camellia sinensis] | *DELLA* | 0.921 |
| CSS0021166 | probable protein phosphatase 2C 8 [Camellia sinensis] | *PP2C* | 0.855 |
| CSS0031264 | probable protein phosphatase 2C 24 [Camellia sinensis] | *PP2C* | 0.957 |
| CSS0037814 | probable protein phosphatase 2C 6 isoform X2 [Camellia sinensis] | *PP2C* | 0.872 |

**Table S4.** Annotation of auxin transport-related genes.

| Gene id | NR annotation | Name |
| --- | --- | --- |
| CSS0008291 | protein PIN-LIKES 7-like isoform X1 [Camellia sinensis] | *PILS5* |
| CSS0041860 | protein PIN-LIKES 7-like [Camellia sinensis] | *PILS7* |
| CSS0006341 | hypothetical protein HYC85_022242 [Camellia sinensis] | *PILS6* |
| CSS0006843 | auxin efflux carrier component 7-like [Camellia sinensis] | *PIN4* |
| CSS0005048 | probable auxin efflux carrier component 1c [Camellia sinensis] | *PIN1* |
| CSS0044702 | auxin efflux carrier component 7-like isoform X1 [Camellia sinensis] | *PIN3* |


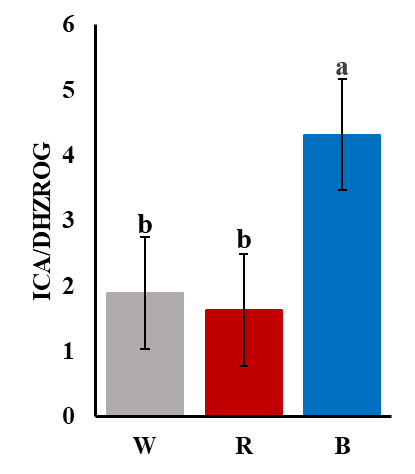


**Figure S1.** Phytohormones ratio. Different letters reveal statistically significant variations (P < 0.05)


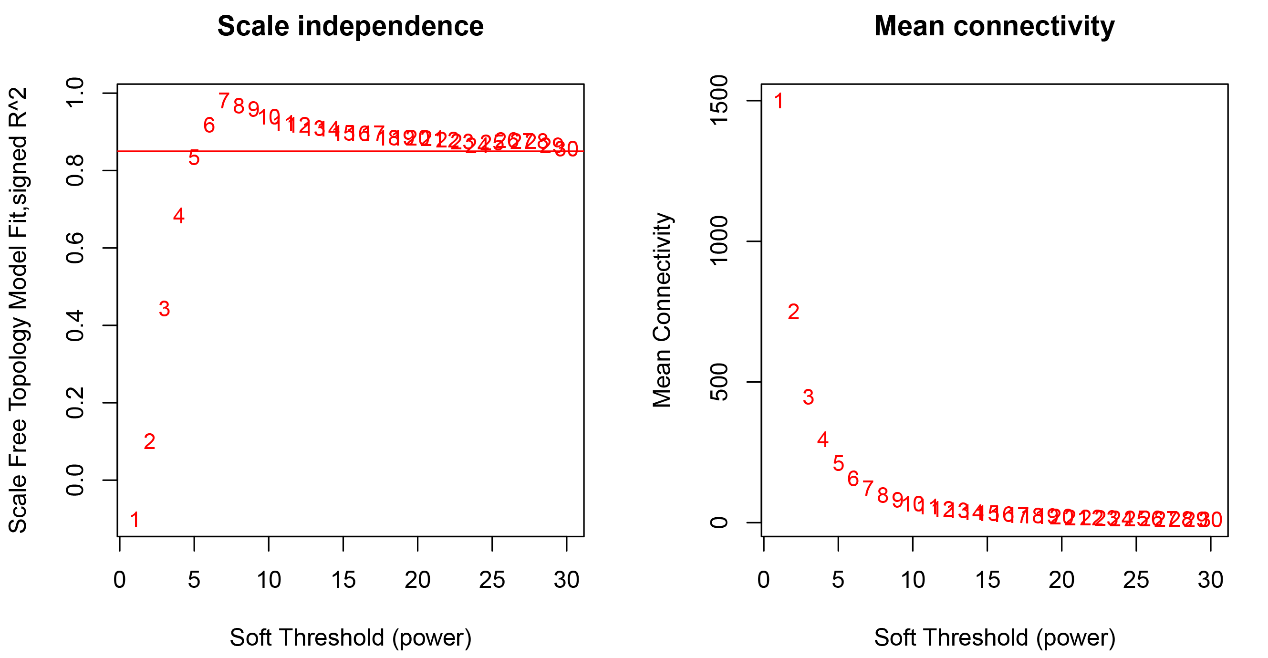


**Figure S2**. Soft-thresholding power for WGCNA.
